# Advanced presentation of cardiac hemangioma

**DOI:** 10.1186/s13019-024-02984-5

**Published:** 2024-11-04

**Authors:** Francesco Rattenni, Francesco Giuseppe Arlati, Andrea Galanti, Fabrizio Sansone, Alberto Clerici, Michele Triggiani, Claudio Muneretto

**Affiliations:** 1 University of Brescia, Brescia, Italy; 2 Manzoni Hospital, Lecco, Italy; 3 Cardio Center De Gasperis, Paediatric Cardiac Surgery, Cardiothoracovascular Department, ASST Grande Ospedale Metropolitano Niguarda, Milan, Italy

**Keywords:** Congenital cardiac tumors, Primary cardiac neoplasms, Cardiac hemangioma, Acute heart failure

## Abstract

Cardiac hemangioma is a rare, benign vascular primary tumor. Clinical presentation is either asymptomatic or with symptoms due to its location and spatial interaction with adjacent structures. The authors present a case of right cardiac hemangioma whose clinical diagnosis was triggered by symptoms of anasarca status, hepatic damage with ascites, pleural effusion and right heart failure. The 79 years-old patient has been treated with complete resection of the tumor by means of cardiopulmonary bypass, without complications. The mid-term outcome (12 months) was favorable. The aim of this study is to report a case of cardiac hemangioma with relevant dimensions, in a rare location (tricuspid valve) with acute onset and interesting aspects of clinical presentation.

## Background

Primary cardiac neoplasms have an incidence of 0.002–2.8% in autopsy series and they can be divided in 75% benign and 25% malignant [1–3]. The most common type of bening tumors is myxoma, which occurs in half of all the benign tumors, then in order of frequency there’s lipoma (19%) and papillary fibroelastoma (17%), instead vascular tumor such as hemangioma is frequent less than 3% and at last there’s hamartoma. Cardiac hemangioma (CH) it’s often found incidentally because of: different location, variability of symptoms and evolution [4, 5]. CH can arise in any cardiac space, they are preferentially located in the right atrium and the presence in the right ventricle is rare [6, 7].

The clinical presentation of CH is non-specific and unrelated to the histological type and dignity of the tumor [8]. It can be either asymptomatic as incidental finding or with symptoms secondary to its spatial relation to adjacent structures: obstructive mechanism, compression of coronary arteries, thromboembolic events. Symptoms will depend on the location, size and growth rate [9]. They can include: arrythmias, pericardial effusion, congestive heart failure, right ventricular outflow tract occlusion, coronary insufficiency, embolization [10–13].

Cardiac hemangioma manifests mostly in the fifth decade and the average age is 43.7 years [7]. We present a case of a 79 years-old patient with an intracardiac mass that growth without clinical manifestation till it became acutely obstructive of the right heart chambers. We performed a successful resection of cardiac hemangioma adherent to the tricuspid valve, manifested with severe obstruction of the right atrio-ventricular junction in an adult.

### Case presentation

A 79 years-old male with chronic atrial fibrillation (AF) presented with a two weeks history of increase in weight and in abdominal circumference, anasarca status associated with dyspnea and shortness of breath at rest (NYHA IV). The patient first episode of AF occurred in 2013, when he had been admitted to the emergency room of a different hospital and an echocardiogram was performed without founding any intracardiac mass. After ten years, he showed acute symptoms and went to the general practitioner who prescribed thoracic radiogram and abdominal ultrasound that showed: lungs congestion with pleural effusion and abdominal ascites with hepatomegaly. Concomitant polycythemia (Haemoglobin 20 g/dL) for which Jak-2 mutation has been searched. Other laboratory findings: NTproBNP 1732, INR 3.5, Creatinin 1.26 mg/dL, Urea 62 mg/dL, WBC 7200 * 10.6 uL, Platelets 193,000 L, AST/ALT 47/26 U/L, bilirubin 2.27 mg/dL, yGT 72 U/L, alkaline fosfatase 221 U/L, lipase 26 U/L, PRC 3 mg/dL, LDH 412 U/L. Arterial blood gas analysis: pH 7.47 pCO2 35.7 pO2 63, SpO2 92, HCO3 26.5 Lactic acid 1.9. ECG showed AF and aspecific anomalies of T wave.

**Fig. 1:**
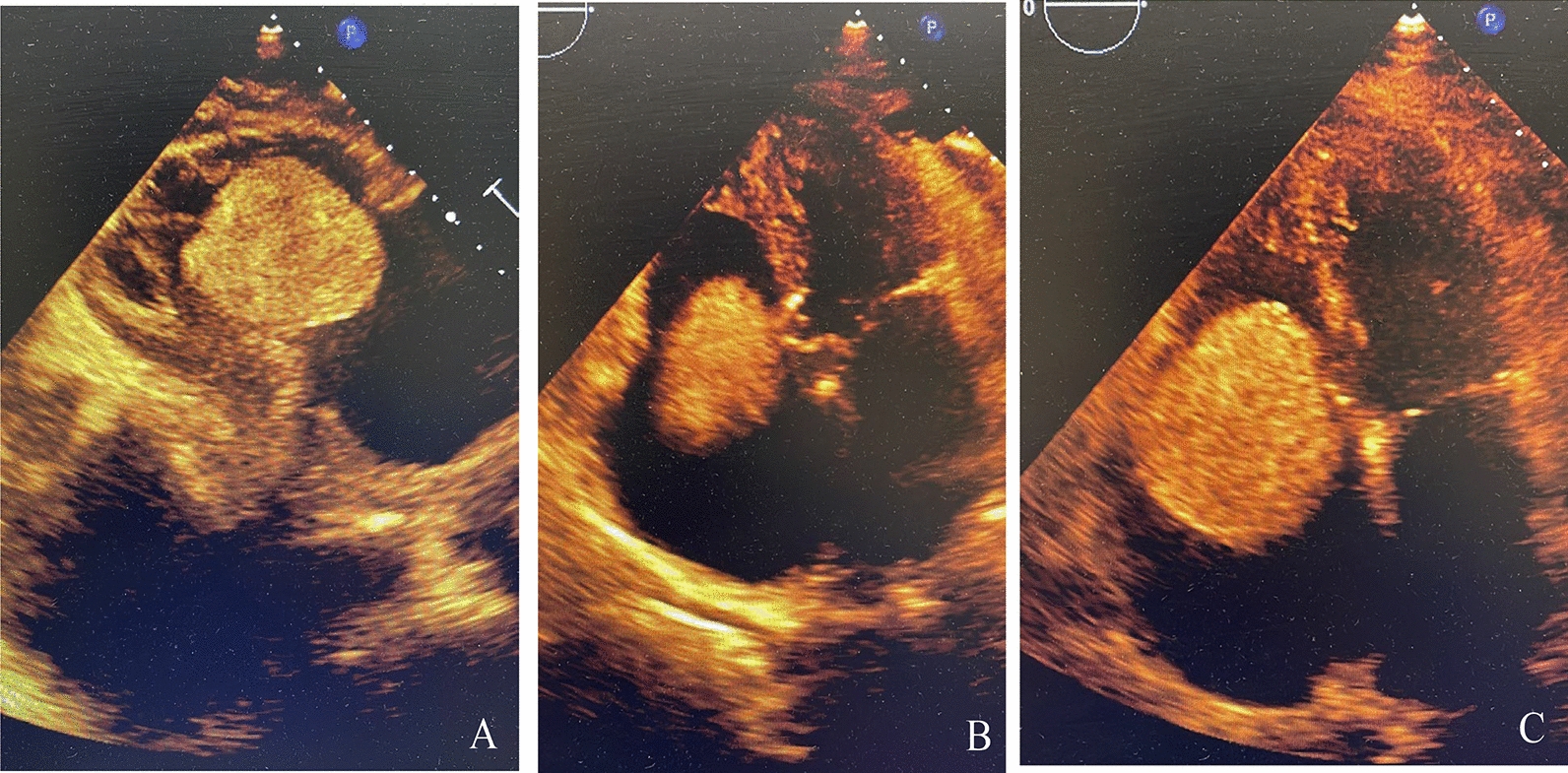
Transthoracic echocardiography of the mass in the right chambers. **A** mass obstructing the tricuspid valve, subcostal view. **B** mass moving back and forth through the tricuspid valve in apical 4 chambers view. **C** Systolic tricuspid valve obstruction in apical 4 chambers view

**Fig. 2:**
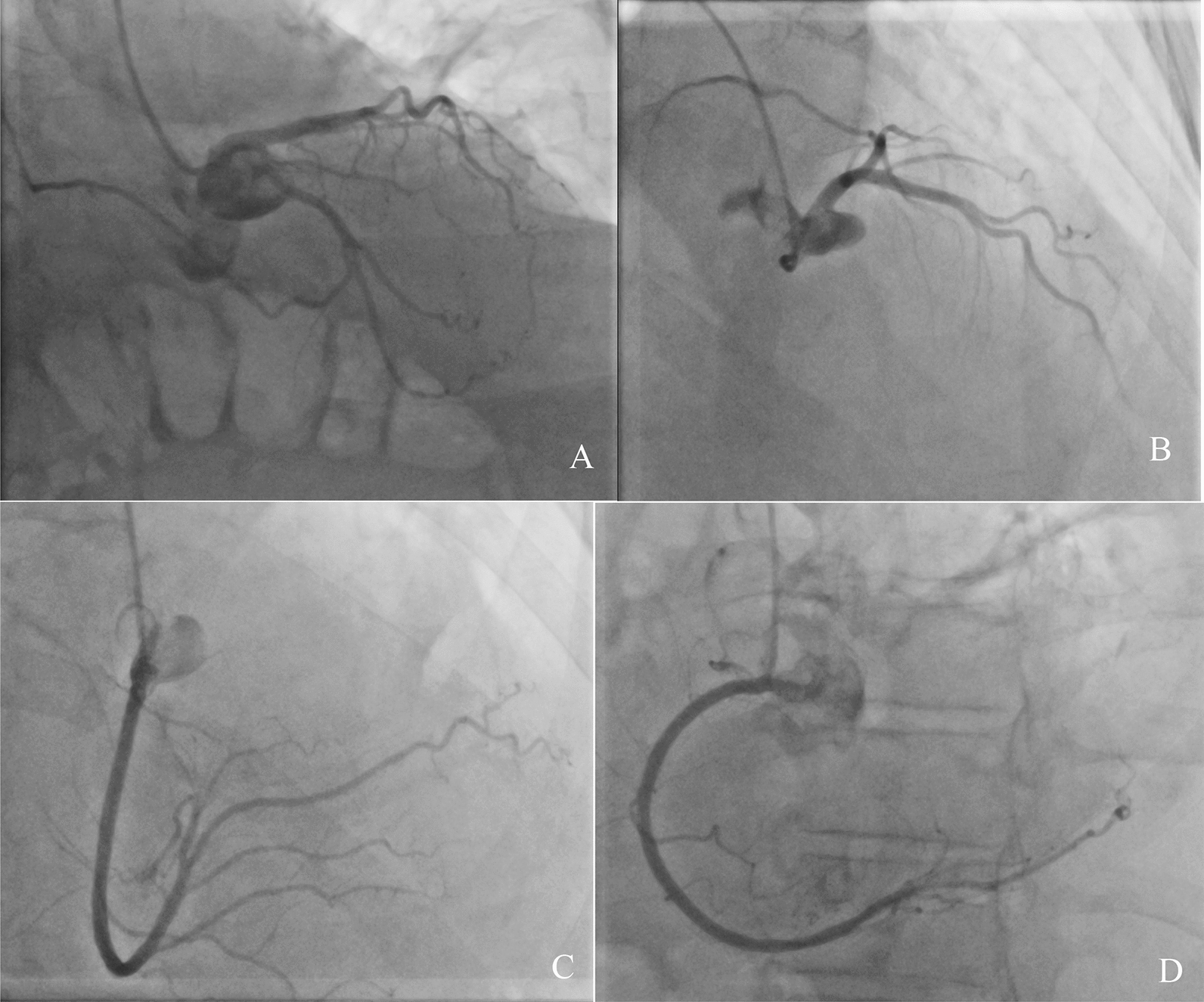
Coronary angiography pre-operative in order to evaluate perfusion of the mass. **A**, **B** left coronary angiogram **C**, **D** right coronary angiogram

The patient has been admitted to the local hospital, where a transthoracic echocardiogram (TTE) revealed an ovoidal hypoechogenic mobile mass of 4.1 × 5.8 cm into the tricuspid valve (Fig. 1A), moving back and forth from the right atrium (Fig. 1B) to the right ventricle (RV) and protruding in the outflow tract (RVOT) (Fig. 1C) in systole, in synchrony with the cardiac cycle. It seemed to originate from the right atrium with a possible pedicle and had a clear border with a normal right ventricular myocardium; no clear blood flow signal was detected in the mass. Right ventricle was dilated, TAPSE reduced (10 mm), right atrium was severely dilated (Vol 71 ml), it wasn’t possible to measure tricuspid gradients and pulmonary pressure. Left ventricle hypertrophy with normal dimension and preserved ejection fraction (EF 60%), mild mitral and aortic valves insufficiency. No pericardial effusion. The patient was referred to our tertiary centre for surgical treatment, with the diagnosis of right cardiac mass conditioning: right heart failure, pleural effusion, anasarca and ascites with hepatic damage. The 3D transthoracic echocardiography (Fig. 1) in our institution confirmed the result of the ETT from the local hospital. Coronarography was performed to exclude coronaropathy and for further evaluation of the eventual vascularization of the mass: we didn’t find a clear perfusion of the mass and it didn’t involve nor compressed the coronary arteries. (Fig. 2A, B: left coronary. Fig. 2C, D: right coronary). Even if we couldn’t totally understand the origin of the mass, it had to be removed with urgence. Euroscore II for urgent single non-CABG operation (surgery on tricupid valve) in this patient: 10.6%.

**Fig. 3:**
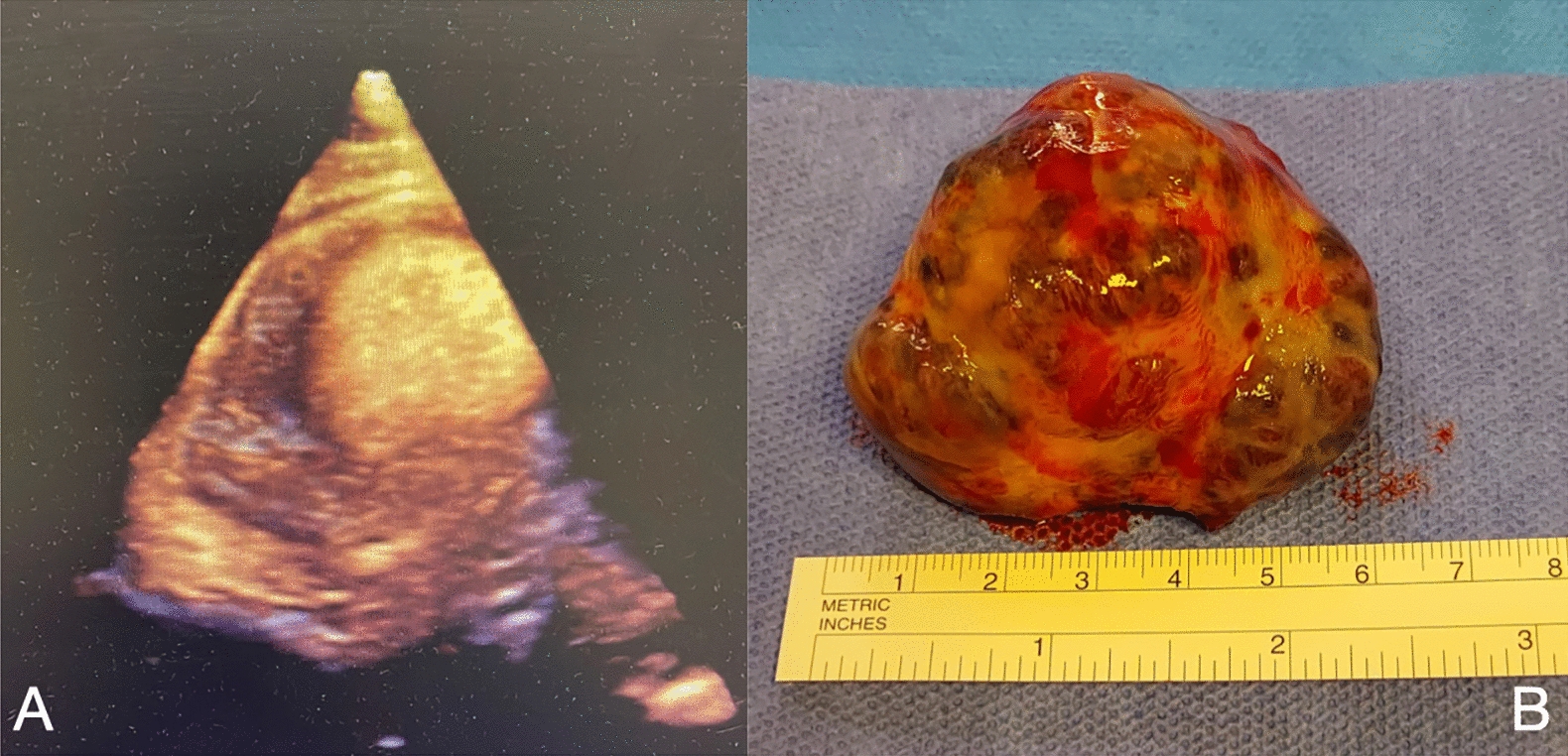
Mass dimensions **A** 3d echocardiography **B** Mass dimensions after the surgical resection (6 centimeters)

After a median sternotomy, cardiopulmonary bypass was instituted with aortic and bicaval cannulation. Following retrograde cardioplegic arrest, we opened the pericardium and the right atrium. We found an oval non-pedunculated purplish heterogeneous mass (50 × 55 mm) adherent to the septal part of the tricuspid valve (Fig. 2B). The mass was excised en-bloc (Fig. 3A) with a capsule dissecting plane and tricuspid valve had consequent massive insufficiency residual, requiring repair with ring annuloplasty implant (Edwards nr. 32).

The intraoperative transesophageal echocardiogram showed no residual lesion and no residual tricuspid insufficiency. The patient needed inotropic support at medium dosage during the initial hours post-operation, was extubated after 2 h with an uneventful postoperative course and discharged on the seventh postoperative day.

The postoperative histopathological diagnosis revealed a benign heterogeneous cardiac hemangioma mixed type: capillary and cavernous. No malignancy was identified. No signs of recurrence of tumor or acute heart failure was observed at 3 months and at 12 months clinical follow-up. The last ETT follow-up showed: normal left ventricle kinesis and dimension, preserved ejection fraction (EF 60%), left and right atrial chambers dilated, mild right ventricular dilatation, TAPSE 12.4 mm, IT moderate (2+), PAPs 40 mmHg.

### Discussion and conclusions

The overall incidence of primary cardiac tumors at autopsy is approximately 17 in a million and cardiac hemangioma constitutes approximately 2.8% of primary cardiac tumors. [3]. CH occurs in the age range from the 20th gestational week [2] to 86 years [3] and its most frequently diagnosed around 44 years [7, 14, 15]. Cardiac hemangioma dimension usually is around 30 mm in diameter, but it has been reported up to 90 mm. Although cardiac neoplasms are often incidental findings, these tumors have the potential to cause significant morbidity and mortality due to their spatial relation to adjacent structures. CH can originate either in the pericardium, myocardium, or endocardium. Mostly, these tumors grow intracavitary, rarely intramurally [16].

In a review on cardiac hemangioma, the location of this tumor was 33.8% in the right ventricle, 32% in the left ventricle, 19% in the right atrium, 8.8% in the left atrium, and 5.8% in the pericardium [17]. Although it may arise from any chamber of the heart, eliciting various clinical symptoms, a review refers that the most common site of right ventricular hemangiomas is the anterior wall of the RV, but only 6.7% are located at the apex of the RV [18]. Cardiac hemangioma such as the one we report that obstructed the right ventricular outflow tract (RVOT) is extremely rare [19].

In terms of physiopathology, hemangioma is a benign tumor that origins from the endothelial cells of the inner layer of blood vessels and it’s highly vascularized. The natural history of CH isn’t clearly defined: it can remain dormant and clinically silent or it may continue to grow, but rarely it’ll undergo spontaneous regression [20].

Most affected patients are asymptomatic, and the pathogenesis of symptoms depends on the tumor’s position and size, causing a wide range of manifestation: dyspnea, arrhythmia, angina, congestive heart failure, right ventricular outflow tract obstruction, pericardial effusion, coronary insufficiency and embolization [5, 6, 11, 21–24]. Few case reports reported a cardiac tumor discovered as a result of congestive heart failure. In these two cases, biopsy confirmed a benign vascular tumor (CH) originating from right ventricle and with obstruction of the RVOT [25, 26].

Regarding tumor location, if hemangioma originates from the myocardium, it can invade the atrioventricular node, resulting in different atrioventricular blocks or myocardial dysfunction [27]. Hemangioma arising from the endocardium tends to expand in the ventricular cavity and cause hemodynamic flow obstruction [23]. The dimension of cardiac hemangioma can range from 20 to 35 mm in diameter, but it has been reported with dimension up to 90 mm [15].

We report a case of a 79 years-old man that became acutely symptomatic. Our patient had been admitted to emergency room in 2013 for AF first presentation, without founding the mass at echocardiogram. After ten years, he presented with shortness of breath and anasarca status, derangements due to congestive heart failure which was due to compression of the tumor tissue (5 × 5 cm) leading to right ventricular occupation and pulmonary blood flow obstruction (Fig. 3A).

The tumor was detected after an echocardiogram. Echocardiography guided the diagnosis toward a cardiac mass but there weren’t other aspects that usually can suggest the hemangioma. Coronary arteriography didn’t show the typical tumor blush: differently from another case report [28], no feeding vessels where originating from the main coronary arteries and where directed clearly toward the tumor [29]. Even on echocardiography there was no obvious blood flow signal in the mass. Enhanced contrast computed tomographic scan or magnetic resonance imaging could establish the diagnosis of hyper-vascularized cardiac tumor, but we didn’t have the pre-operation time to make ulterior imaging evaluation [30, 31]. In certain cases, cardiac catheterization and an angiogram have been able to establish a diagnosis of cardiac hemangioma by signs of an intracavity filling defect on ventricular angiogram and vascular blush on coronary arteriography. Also enhanced-contrast MRI and computed tomography can suggest the specific characteristics of tumor [19, 32].

In our case report the preservation of tricuspid valve with its repair with a ring has been possible: in another recent case report, the cardiac hemangioma was attached to the anterior leaflet of tricuspid valve and the head of the anterior papillary muscle. This required the complete replacement of the valve with a 31-mm bioprosthetic valve in order to remove completely the mass [33].

Based on morphology, hemangioma can be either capillary (small vessels), cavernous (multiple dilated thin-walled vessels), or arteriovenous (dysplastic malformed arteries and veins) [15]. A combination of these three subtypes is often reported, and in our case, CH had elements of both the capillary and cavernous subtypes.

Management of right ventricular tumors remains a challenging topic, due to their rarity and few clinical evidences. The treatment approach depends on several factors: tumor size, symptoms, growth pattern and potentially risk complications [33] .

The most reported and standard treatment in literature is curative surgical resection, even if conservative management has been reported [34–36] The first surgical resection of CH was described in 1960 [37]. Surgical treatment operative mortality rate of 2%, depending principally from tumor histology as a most significant predictor of mortality [38]. Generally long-term prognosis after surgical resection is extremely favorable and most patients do not have a recurrence of the tumor [19].

Conservative management with an alternative approach has been reported: medical treatment with beta-blockers such as propranolol, typically utilized in skin hemangioma, can lead to successful decrease in tumor volume [35, 36]. However, its efficacy and long-term outcome is uncertain.

In term of prognosis, current available data aren’t enough to conclude on the benefit of medical therapy versus curative surgery, leaving by now the medical option as a second choice in tumors which can’t be completely removed.

As far as we know, complete tumor resection is crucial for curative treatment, even if natural history of ventricular hemangioma is not completely understood. The last option for complete tumor resection as treatment of primary invasive cardiac tumors is orthotopic heart transplantation [39, 40].

In our case, the patient had uneventful postoperative course and was discharged on the seventh postoperative day. The patient was free of recurrence after surgical resection with no heart dysfunction observed at the three- and twelve-months postoperative follow-up.

## Data Availability

No datasets were generated or analysed during the current study.
